# Production of a microcapsule agent of chromate-reducing *Lysinibacillus fusiformis* ZC1 and its application in remediation of chromate-spiked soil

**DOI:** 10.1186/s40064-016-2177-6

**Published:** 2016-05-04

**Authors:** Jun Huang, Jingxin Li, Gejiao Wang

**Affiliations:** State Key Laboratory of Agricultural Microbiology, College of Life Science and Technology, Huazhong Agricultural University, Wuhan, 430070 People’s Republic of China

**Keywords:** Chromate-reducing bacterium, *Lysinibacillus fusiformis*, Medium optimization, Microcapsule agent, Spray drying, Soil bioremediation

## Abstract

*Lysinibacillus fusiformis* ZC1 is an efficient Cr(VI)-reducing bacterium that can transform the toxic and soluble chromate [Cr(VI)] form to the less toxic and precipitated chromite form [Cr(III)]. As such, this strain might be applicable for bioremediation of Cr(VI) in soil by reducing its bioavailability. The study objective was to prepare a microcapsule agent of strain ZC1 for bioremediation of Cr(VI)-contaminated soil. Using a single-factor orthogonal array design, the optimal fermentation medium was obtained and consisted of 6 g/L corn flour, 12 g/L soybean flour, 8 g/L NH_4_Cl and 6 g/L CaCl_2_. After enlarged fermentation, the cell and spore densities were 5.9 × 10^9^ and 1.7 × 10^8^ cfu/mL, respectively. The fermentation products were collected and embedded with 1 % gum arabic and 1 % sorbitol as the microcapsule carriers and were subsequently spray-dried. Strain ZC1 exhibited viable cell counts of (3.6 ± 0.44) × 10^10^ cfu/g dw after 50-day storage at room temperature. In simulated soil bioremediation experiments, 67 % of Cr(VI) was reduced in 5 days with the inoculation of this microcapsule agent, and the Cr(VI) concentration was below the soil Cr(VI) standard level. The results demonstrated that the microcapsule agent of strain ZC1 is efficient for bioremediation of Cr(VI)-contaminated soil.

## Background

Chromium (Cr) is one of the most abundant heavy metals and primarily presents in the natural environment in chromite [Cr(VI)] and chromate [Cr(III)] forms (Srivastava and Thakur 2007; Liu et al. 2012). The Cr(VI) form is much more toxic and soluble than the Cr(III) form, and Cr(III) exhibits greater affinity for organics and consequently forms insoluble complexes (Nickens et al. 2010). Because of its mutagenic and carcinogenic characteristics, Cr(VI) and its compounds are recognized by the United States Environmental Protection Agency as priority pollutants (USEPA 1996). It is known that Cr(VI) enters the cell through the sulfate transport channels, possibly due to the structural similarity between Cr(VI) and sulfate (Ramírez-Díaz et al. 2008). After entering the cell, Cr(VI) generates reactive oxygen species (ROS), which can cause DNA damage and oxidative deterioration of lipids and proteins (Bagchi et al. 2002; Liu et al. 2014; Ackerley et al. 2004; Valko et al. 2005; Wang et al. 2006).

Currently, Cr(VI) contamination is increasing rapidly and is widespread in soil, aquatic sediment, surface water and groundwater due to the extensive use of Cr in various industrial processes (Yang et al. 2009; Cheng et al. 2012). A total of 450,000 tons of Cr(VI)-containing wastes are discharged per year in China (Gao and Xia 2011). The extensively widespread Cr(VI) form could be taken up into the food chain and eventually cause a series of human health risks (Nickens et al. 2010). In addition, Cr(VI) also alters the microbial community structure and activity in soil (Shi et al. 2002; Desai et al. 2009). Hence, an urgent need exists for remediation of Cr(VI)-contaminated soil. Traditional technologies for Cr(VI) remediation include chemical reduction (Su and Ludwig 2005), solidification/stabilization (Kumpiene et al. 2008) and electrokinetic remediation (Lu et al. 2012). However, these methods suffer from a number of problems, such as high cost, low efficiency, consumption of energy and chemical reagents, and generation of secondary environmental pollution (Dhal et al. 2013). In contrast, biological remediation has attracted increasing attention due to its low environmental impact and low-cost advantages (Wu et al. 2010).

Bioremediation of Cr(VI) compounds involves different strategies, such as biosorption, bioaccumulation and bioreduction (Srinath et al. 2002). Microbial reduction of Cr(VI) to less toxic and less mobile Cr(III) is considered an effective method for remediation of Cr(VI)-contaminated soil (Kanmani et al. 2012). Certain microorganisms have been reported to reduce Cr(VI) in soil, such as strains of *Pseudomonas* (Oves et al. 2013), *Microbacterium* (Soni et al. 2014), *Brucella* (Maqbool et al. 2015), *Bacillus* (He et al. 2010; Kathiravan et al. 2011; Kumari et al. 2014), *Streptomyces* (Polti et al. 2009; Aparicio et al. 2015), *Staphylococcus* (Zhang et al. 2014) and *Pannonibacter phragmitetus* (Chai et al. 2009; Liao et al. 2014; Wang et al. 2015). However, most of the studies on bioreduction of Cr(VI) were performed with direct addition of pure cultures to soil, which is inconvenient for transportation and storage. Currently, few studies have investigated the possibility of producing a microcapsule microbial agent for bioremediation of Cr(VI)-contaminated soil. It is known that a microcapsule reagent has the ability to retain the physical characteristics of substances and is less sensitive to temperature, light, oxygen and humidity (Desai and Park 2005; Sabikhi et al. 2010). Microcapsule reagents could enhance the biological activity of several biological control agents and protect them from adverse environments (Jin and Custis 2011). Therefore, bioremediation using a microcapsule microbial agent is a promising method for better use of Cr(VI)-reducing strains.

*Lysinibacillus fusiformis* ZC1 is a highly Cr(VI) resistant strain that can efficiently reduce Cr(VI) to Cr(III) (He et al. 2011). Because Cr(III) is less soluble and less bioavailable, such a strain might be applicable to immobilization of Cr(VI) in soil by causing plants to adsorb less Cr. Previously, we found that the growth of tobacco in Cr(VI)-containing pot experiments was promoted, and the Cr(VI) contents in roots and leaves were reduced with the addition of fresh ZC1 culture (Jia et al. in preparation). The objective of this study was to produce a microcapsule agent of strain ZC1 at low cost and with ease of manipulation for bioremediation of Cr(VI) spiked soil. The culture conditions were optimized using an orthogonal test, and the microcapsule agent was obtained by spray drying after fermentation. The remediation efficiency of Cr(VI)-contaminated soil with this microcapsule agent was significantly increased in simulated soil microcosm Cr(VI) remediation experiments. The current findings present a portable and effective method to produce a microcapsule microbial agent for bioremediation of Cr(VI)-contaminated soil.

## Results

### Selection of medium components

**Fig. 1:**
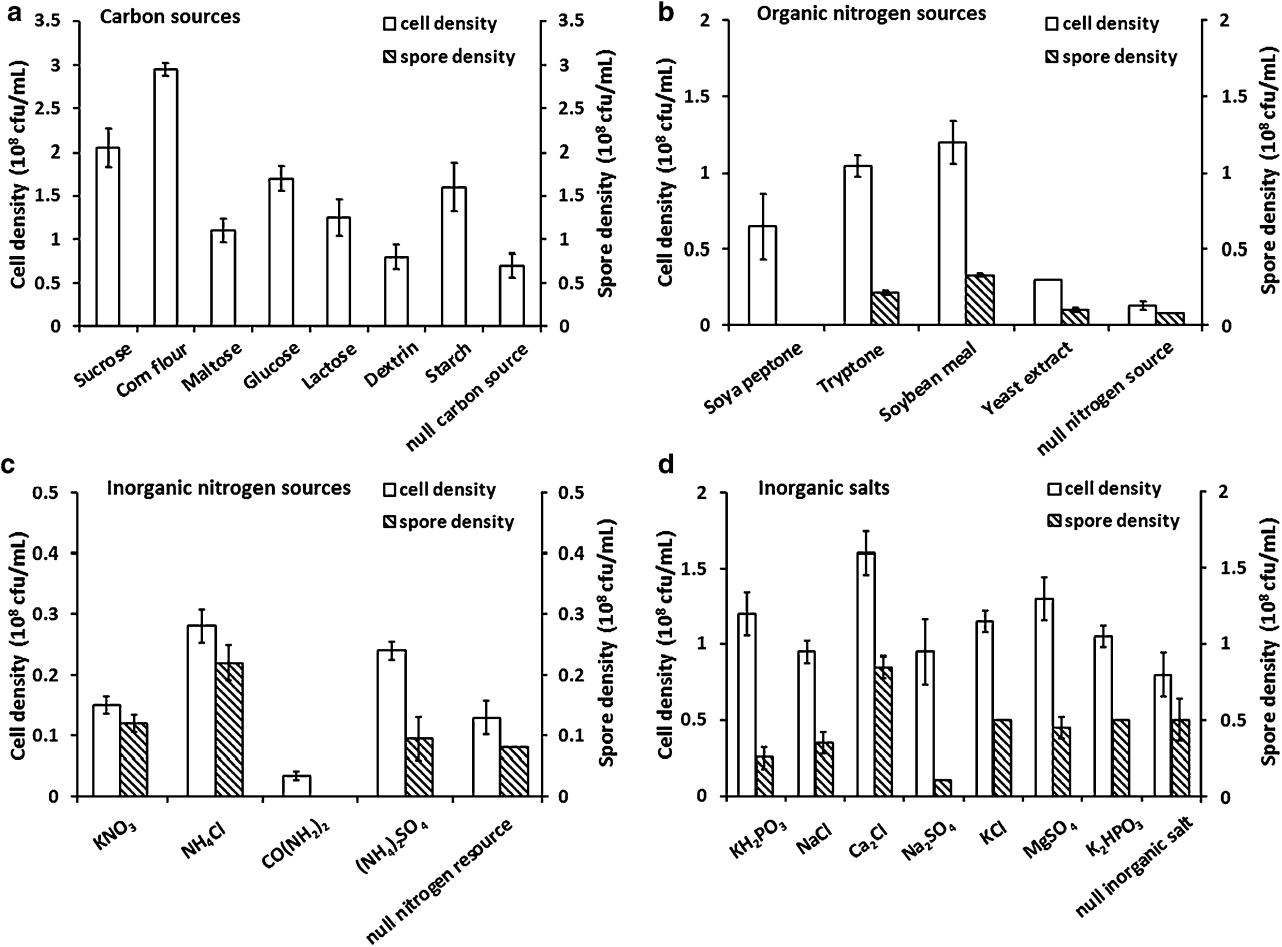
Effects of different nutritional components on *L. fusiformis* ZC1 cell and spore density. **a** Carbon sources, including sucrose, corn flour, maltose, glucose, lactose, dextrin and starch. Null carbon source indicates medium containing 5 g/L tryptone, 3 g/L yeast extract, 6 g/L KH_2_PO_3_ and absence of a carbon source. **b** Organic nitrogen sources including soya peptone, tryptone, soybean meal and yeast extract. Null nitrogen source indicates medium containing 2 g/L corn flour, 6 g/L KH_2_PO_3_ with absence of a nitrogen source. **c** Inorganic nitrogen sources, including KNO_3_, NH_4_Cl, CO(NH_2_)_2_ and (NH_4_)_2_SO_4_. **d** Inorganic salts, including KH_2_PO_3_, NaCl, CaCl_2_, Na_2_SO_4_, KCl, MgSO_4_ and K_2_HPO_3_. Null inorganic salt indicates medium containing 2 g/L corn flour, 8 g/L soybean meal, and 8 g/L NH_4_Cl without an inorganic salt

To optimize medium cost with ideal cell and spore production, single-factor experiments were performed for selection of the culture medium components. Different carbon sources, organic nitrogen sources, inorganic nitrogen sources and inorganic salts were considered for strain ZC1 growth and spore production. Seven carbon sources were investigated, i.e., sucrose, corn flour, maltose, glucose, lactose, dextrin and starch. Among the various carbon sources tested, corn flour had the most prominent effect on the growth of strain ZC1, reaching (2.95 ± 0.07) × 10^8^ cfu/mL cells (Fig. 1a). The basal medium contained 5 g/L tryptone, 3 g/L yeast extract and 6 g/L KH_2_PO_3_.

To further optimize the culture medium components, the effects of nitrogen sources were studied, including organic nitrogen sources (soya peptone, tryptone, soybean flour, yeast extract) and inorganic nitrogen sources [KNO_3_, NH_4_Cl, CO(NH_2_)_2_, (NH_4_)_2_SO_4_]. Strain ZC1 was inoculated in basal medium containing 2 g/L corn flour, 6 g/L KH_2_PO_3_ and various nitrogen sources. It was shown that the highest cell and spore production rates were achieved in medium supplemented with soybean flour and NH_4_Cl, respectively (Fig. 1b, c).

Additionally, seven inorganic salts of KH_2_PO_3_, NaCl, CaCl_2_, Na_2_SO_4_, KCl, MgSO_4_ and K_2_HPO_3_, were chosen to examine the effects of the different inorganic salts on viable cell and spore productions. Each inorganic salt was added to basal medium containing 2 g/L corn flour, 8 g/L soybean flour and 8 g/L NH_4_Cl. As shown in Fig. 1d, CaCl_2_ resulted in the highest cell and spore productions. Therefore, corn flour, soybean flour, NH_4_Cl and CaCl_2_ were selected as the best components for further investigation.

### Optimization of concentrations of the medium components

An orthogonal array design L_9_(3^4^) was used to optimize the concentration of each culture medium component. The factor levels in the orthogonal experiment are listed in Table 1. The final experiment results and the effects of those factors on the yield of strain ZC1 cells and spores are shown in Table 2. According to the magnitude of k or K (Table 2), the optimal combinations for viable cell and spore productions were A3B3C2D1 and A3B2C2D2, respectively.

**Table 1:** Factors and their levels in the orthogonal experiment L_9_(3^4^)

Constituent	Symbol	Concentration (g/L)		
		Level 1	Level 2	Level 3
Corn flour	A	2	4	6
Soybean flour	B	4	8	12
NH_4_Cl	C	4	8	12
CaCl_2_	D	3	6	9

**Table 2:** Experimental design and results of the orthogonal experiment

Run	A (g/L)	B (g/L)	C (g/L)	D (g/L)	Cell density (10^8^ cfu/mL)	Spore density (10^8^ cfu/mL)
1	2	4	4	3	1.00^a^	0.25^a^
2	2	8	8	6	1.65	0.7
3	2	12	12	9	1.60	0.1
4	4	4	8	9	1.50	0.2
5	4	8	12	3	1.85	0.45
6	4	12	4	6	1.90	0.4
7	6	4	12	6	2.00	0.85
8	6	8	4	9	2.40	0.8
9	6	12	8	3	3.00	0.55
k1^b^	1.42	1.50	1.77	1.95		
k2	1.75	1.97	2.05	1.85		
k3	2.47	2.17	1.82	1.83		
r^c^	1.05	0.67	0.28	0.24		
Optimal level	3	3	2	1		
K1^b^	0.33	0.43	0.48	0.42		
K2	0.35	0.65	0.48	0.65		
K3	0.73	0.33	0.35	0.35		
R^c^	0.4	0.32	0.13	0.3		
Optimal level	3	2	2	2		

^a^Values indicate means of three replicates

^b^k_i_^A^ is the mean of ∑cell density at A_i_, K_i_^A^ is the mean of ∑spore density at A_i_

^c^r^A^ = max(k_i_^A^) − min(k_i_^A^), R^A^ = max(K_i_^A^) − min(K_i_^A^)

The variance was calculated to evaluate the significance of each culture component. The results indicated that corn flour (A) and soybean flour (B) both had significantly positive effects on strain ZC1 viable cell and spore productions, NH_4_Cl (C) had a slightly positive effect only on viable cell production, and CaCl_2_ (D) had a significantly positive effect only on spore production (Table 3). Therefore, to maximize both viable cell and spore productions, the theoretical optimized combination should be A3B3C2D2, and an additional growth experiment was performed out to confirm this choice. The results illustrated that the cell and spore densities of A3B3C2D2 were 3.3 × 10^8^ and 0.68 × 10^8^ cfu/mL, respectively, which are higher than those of A3B3C2D1 and A3B2C2D2.

**Table 3:** Analysis of variance for experimental results

Source	Sum of squares	Degree of freedom	Mean square	F value	*p* value
For cell density					
Corn flour	1.727	2	0.864	72.302	0.014*
Soybean flour	0.702	2	0.351	29.395	0.033*
NH_4_Cl	0.137	2	0.069	5.744	0.148
Error	0.024	2	0.012		
Total	2.591	8			
For spore density					
Corn flour	0.294	2	0.147	529.000	0.002**
Soybean flour	0.144	2	0.072	259.000	0.004**
CaCl_2_	0.137	2	0.069	247.000	0.004**
Error	0.001	2			
Total	0.576	8			

* *p* < 0.05; ** *p* < 0.001

Thus, the optimal concentrations of the four components were confirmed as 6 g/L corn flour, 12 g/L soybean flour, 8 g/L NH_4_Cl and 6 g/L CaCl_2,_ respectively. The optimal medium produces a cell yield similar to that of the original LB medium but is much less expensive (He et al. 2011).

### Bioreactor fermentation

**Fig. 2:**
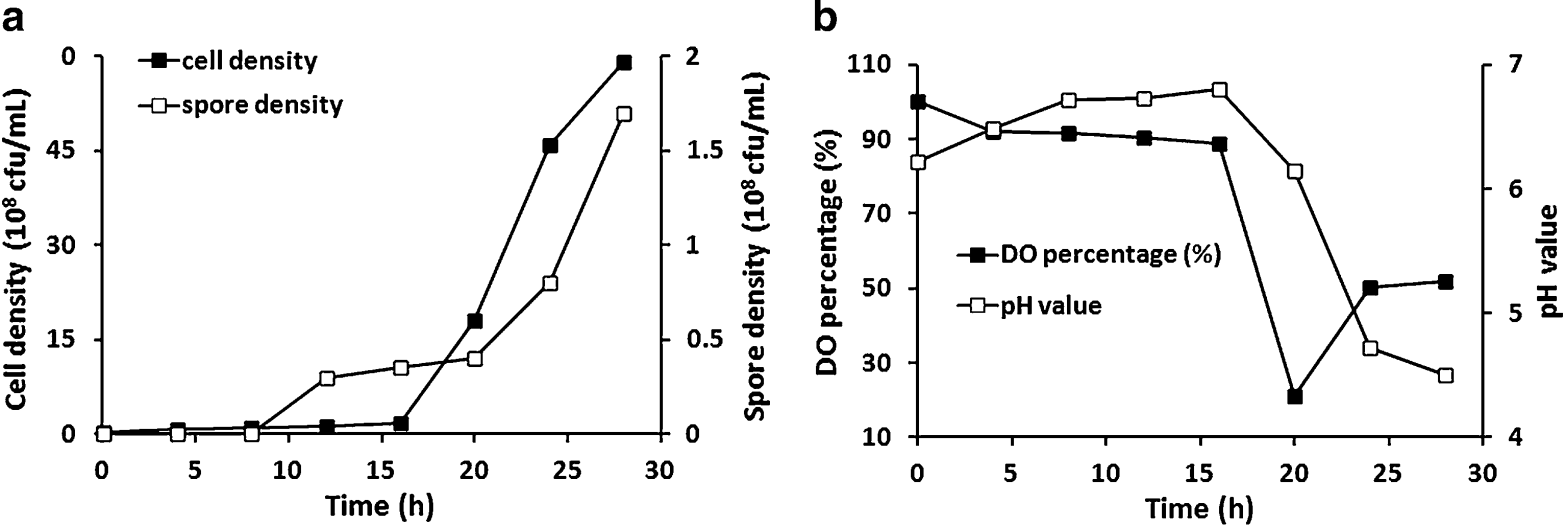
Time course of fermentation using optimized medium for *L. fusiformis* ZC1 in a 50 L fermenter. **a** Curves of cell (*filled box*) and spore (*open box*) density during fermentation process. **b** Curves of DO percentage (*filled box*) and pH value (*open box*) during fermentation process

A fermentation process using the optimal medium was performed in a 50 L fermenter. Based on the characteristics of strain ZC1, the fermentation process was conducted under the following conditions: inoculum volume of 2 % (v/v), cultivation temperature at 37 °C, initial pH of 8.0 and cultivation time of 28 h (early stationary phase). As shown in Fig. 2a, the numbers of viable cell and spore production were increased rapidly after 16 h, at which time strain ZC1 grew into the log phase. At the same time, the pH and dissolved oxygen values were decreased sharply from 16 h to 20 h (Fig. 2b). The counts of strain ZC1 cells and spores reached maximum of 5.9 × 10^9^ and 1.7 × 10^8^ cfu/mL, respectively, at 28 h. The fermentation was terminated when the pH value declined to 4.5, which was not suitable for continuous growth of strain ZC1.

### Production of microcapsule agent by spray drying and storage

**Fig. 3:**
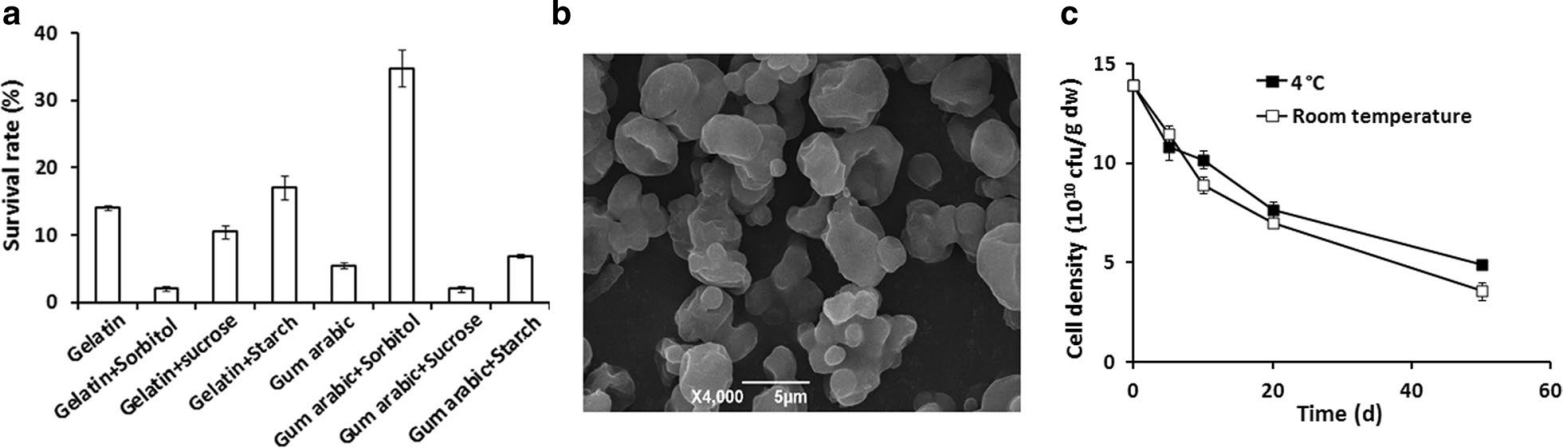
Spray drying of *L. fusiformis* ZC1 microcapsule with different carriers and storage. **a** Survival rate of *L. fusiformis* ZC1 with different carriers spray-dried at an inlet/outlet temperature setting of 160/70 °C. **b** SEM micrographs of *L. fusiformis* ZC1 microcapsule with 1 % (w/v) gum arabic and sorbitol (1:1). **c** Growth curves of viable cell counts of microcapsule agents containing *L. fusiformis* ZC1 microcapsule with 1 % (w/v) gum arabic and sorbitol (1:1) under storage at 4 °C (*filled box*) and room temperature (*open box*) conditions

Subsequent efforts focused on production of a microcapsule agent using the fermentation products, the survival rates of strain ZC1 with different carriers in the process of spray drying were investigated. The results demonstrated that strain ZC1 acquired the highest survival rate of (34.8 ± 2.75) % with a combination of 1 % (w/v) gum arabic and sorbitol (1:1), which yielded viable cell counts of (1.4 ± 0.11) × 10^11^ cfu/g dw (Fig. 3a). Hence, the most appropriate carrier for strain ZC1 was the combination of 1 % (w/v) gum arabic and 1 % sorbitol (1:1).

To investigate the protective efficacy of the carriers, scanning electron microscopy (SEM) was used to observe the micromorphology of the spray-dried microcapsule agent. It was shown that the resulting particles were nearly spherical and dispersed uniformly with similar sizes from 1 to 10 μm (Fig. 3b). The microcapsule particles had smooth surfaces with no apparent cracks or pores, which could decrease the permeability of gases and better protect the core. The results indicated that bacterial cells of strain ZC1 were successfully microcapsule with the carriers. Hence, the combination of 1 % (w/v) gum arabic and sorbitol (1:1) as carriers yielded the highest survival rate and also protected the cells quite well.

The spray-dried microcapsule agent particles were stored in sealed bags made of aluminum–polyester–polyethylene, and the stabilities were investigated at 4 °C and room temperature. The results showed that the viable cell counts of the microcapsule agents decreased slowly during storage under both temperature conditions (Fig. 3c). However, (3.6 ± 0.44) × 10^10^ cfu/g dw cells survived in the microcapsule agent after 50-day storage at room temperature, which was quite similar to that of storage at 4 °C [(4.9 ± 0.21) × 10^10^ cfu/g dw] (Fig. 3c). The changes in viable cell counts for the microcapsule agents stored at both 4 °C and room temperature conditions are shown in Table 4. No statistically significant differences (*p* < 0.05) were observed in the number of viable cell counts of the microcapsule agents for those stored at 4 °C and room temperature, indicating that the survival of the microcapsule agent was not strictly tied to temperature.

**Table 4:** Dynamic changes in viable cell counts of the microcapsule agent during the storage period

Storage time (days)	Viable cell counts (10^10^ cfu/g dw)		F value	*p* value
	4 °C	Room temperature		
0	13.9 ± 1.1	13.9 ± 1.1	0.000	1.000
5	10.8 ± 0.6	11.46 ± 0.4	0.693	0.493
10	10.2 ± 0.4	8.9 ± 0.4	4.500	0.168
20	7.6 ± 0.4	7.0 ± 0.2	1.800	0.312
50	4.9 ± 0.2	3.6 ± 0.4	11.376	0.078

* *p* < 0.05; ** *p* < 0.001

### Simulation of Cr(VI) soil microcosm remediation

**Fig. 4:**
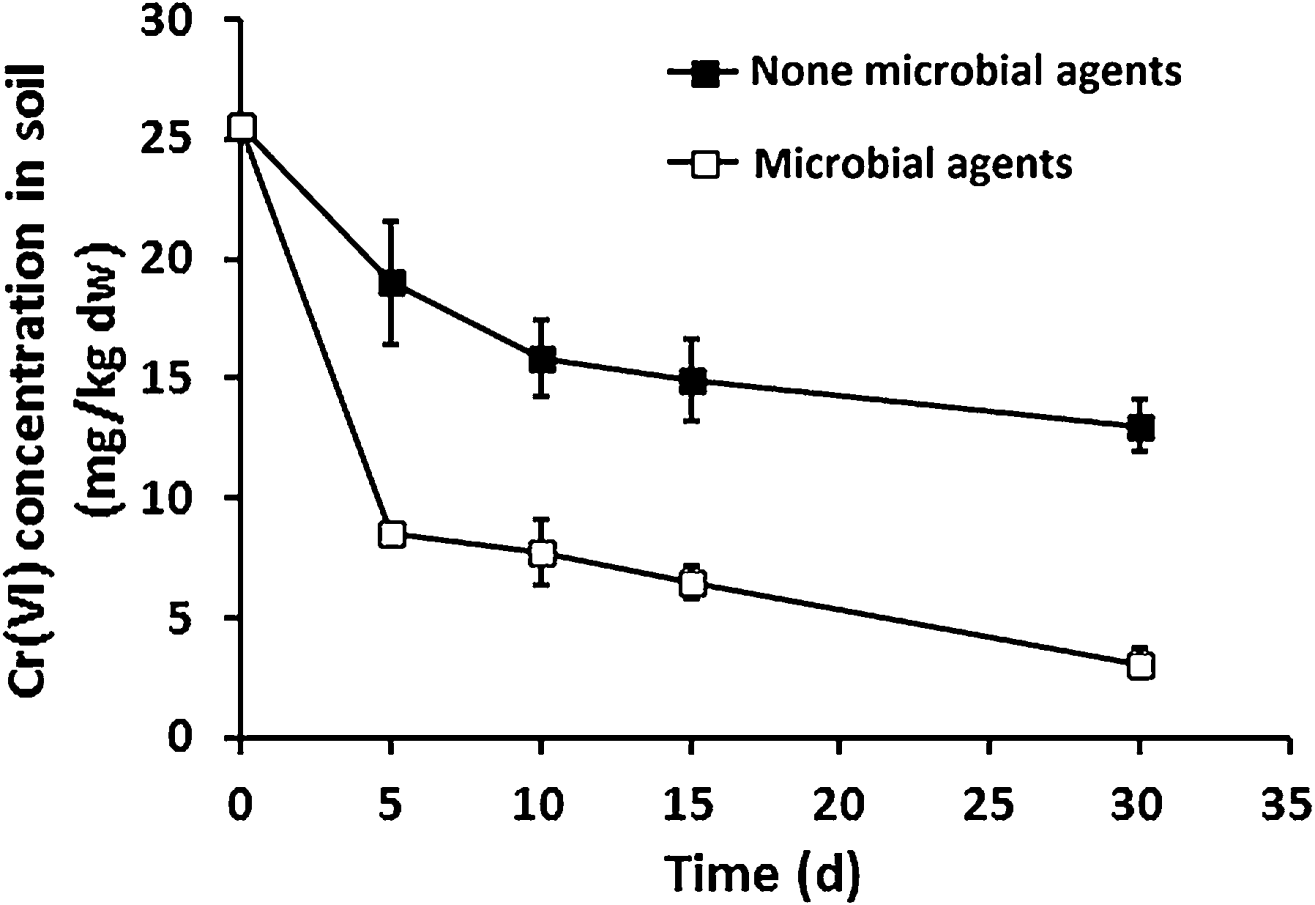
Cr(VI) reduction from contaminated soil by the microcapsule agent of *L. fusiformis* ZC1. Dynamic changes of Cr(VI) concentration after addition of 28 mg/kg Cr(VI) dw to soil, amended with the microcapsule agent (*open box*), and absent the microcapsule agent (*filled box*)

Bioreduction of Cr(VI) to the less toxic and less bioavailable Cr(III) is considered an effective approach for bioremediation of Cr(VI)-contaminated soil. To study the bioreduction efficiency of the microcapsule agent of strain ZC1, a simulated soil microcosm remediation experiment was conducted at laboratory scale. The soils used in this study were artificially mixed with 28 mg/kg dw Cr(VI), which corresponds to three times the soil Cr(VI) permissible level (9 mg/kg dw) (Kumari et al. 2014). With microcapsule agent inoculation, 67 % Cr(VI) was reduced into Cr(III) in the first 5 days, which was significantly better than that of uninoculated control soils (25.6 %). In this case, the Cr(VI) concentration of soils with the addition of the microcapsule agent was reduced to (8.5 ± 0.1) mg/kg dw after 5-day bioreduction, which was lower than the soil Cr(VI) permissible level. In contrast, the Cr(VI) concentration of uninoculated control soils was reduced to (19.0 ± 2.6) mg/kg dw, which was still two times higher than the permissible level. Ultimately, 88 % Cr(VI) was eventually reduced into Cr(III) in 30 days under the effect of microcapsule agent, which is 48.93 % higher than that of the uninoculated control soils (Fig. 4). These results indicated that the microcapsule agent of strain ZC1 has a remarkable effect on the bioreduction of Cr(VI) in soil.

## Discussion

Previously, we reported that *L. fusiformis* ZC1 is an efficient Cr(VI)-reducing strain during cultivation with LB medium. This strain could resist up to 60 mM Cr(VI) and reduce 1 mM Cr(VI) completely within 12 h in LB medium (He et al. 2011). In addition, we found that the growth of tobacco was promoted in Cr(VI)-containing pot experiments, and the Cr(VI) contents in roots and leaves were reduced with the addition of fresh ZC1 culture (Jia et al. in preparation). To apply this strain more efficiently and with low cost, in this study, we focused on medium preparation with low cost and production of a stable microcapsule agent.

It has been reported that the carbon sources, mixed nitrogen source and inorganic salts are important for bacterial growth and spore productions (Rao et al. 2007; Shi and Zhu 2007; Chen et al. 2010; Sella et al. 2009). Hence, the culture medium of strain ZC1 was optimized for low cost and high yields of bacterial cells and spores. In optimization of the culture medium, the combination of a single-factor experiment and orthogonal array design proved to be a powerful tool. The optimized medium containing 6 g/L corn flour, 12 g/L soybean flour, 8 g/L NH_4_Cl and 6 g/L CaCl_2_ offers a price advantage and a cell yield similar to that of the traditional LB medium (He et al. 2011).

Spray drying is the most common technique for microencapsulation of microbes from fermentation broth because of its low cost and high capacity, which makes it suitable for large-scale manufacture (Sunny-Roberts and Knorr 2009). Factors that affect the bacterial survival rate during spray drying are grouped into intrinsic (stress tolerance) and extrinsic factors (including growth phase, drying conditions and carrier materials) (Fu and Chen 2011). *L. fusiformis* ZC1 is a Gram-positive bacterium with a thicker cell wall that displays higher stress and heat tolerance during the process of spray drying. In addition, it was reported that in *E. coli* K12 and *L. acidophilus* strains, the survival rates of bacterial cells harvested in the early-stationary phase were much higher than those in the mid-log phase (Pispan et al. 2013; Morgan et al. 2006). In this study, the bacterial cells of strain ZC1 were harvested at the early-stationary phase, which is consistent with previous studies.

To find a suitable protective carrier for the production of a microcapsule agent, the carriers mentioned in the previous studies were selected, including sucrose, starch, sorbitol, gum arabic and gelatin (Hernandez et al. 2007; Fu and Chen 2011; Salar-Behzadi et al. 2013). The results indicated that the viability of strain ZC1 could be well protected by microencapsulation with 1 % gum arabic and sorbitol (1:1) during spray drying at elevated temperatures. Different from *Trichoderma* sp. which used chlamydospores and conidia as the active ingredients in most products, *L. fusiformis* mainly used nutritive cells as the active ingredients (Jin and Custis 2011; He et al. 2011). The viable cell count of the microcapsule agent of strain ZC1 was (1.4 ± 0.11) × 10^11^ cfu/g dw, which was significantly higher than that of *Trichoderma* sp. (Harman and Custis 2006).

Compared with chilled or frozen preservation, a microcapsule agent stored in a vacuum or under nitrogen has the advantages of low cost and high stability for long-term storage (Hernandez et al. 2007). The spray-dried microcapsule agent of strain ZC1 was stored in vacuum-sealed bags of aluminum–polyester–polyethylene, thus avoiding oxygen, moisture, light and microbial contamination (Morgan et al. 2006). The viable cell counts at 4 °C and room temperature were quite similar with values of (4.9 ± 0.21) × 10^10^ and (3.6 ± 0.44) × 10^10^ cfu/g dw after 50-day storage. Such cell numbers are still satisfactory for bioremediation purposes. In addition, no statistically significant differences (*p* < 0.05) were observed between the viable cell counts at two different temperature conditions during the entire storage period. Therefore, storage of microcapsule agent of strain ZC1 at room temperature is more convenient and economical.

Bioreduction has been found to be an useful method for reducing Cr(VI) to insoluble and nontoxic Cr(III) in soil, which could decrease Cr(VI) accumulation in plants and significantly improve the quality of Cr(VI)-contaminated soil (Kumari et al. 2014; Jeyalakshmi and Kanmani 2008). Various studies have demonstrated the effectiveness of the microbes in bioremediation of Cr(VI)-contaminated soil, but most of these studies were performed in batch reactors or conducted by adding pure cultures directly to soil (Ge et al. 2015; Kumari et al. 2014; Xiao et al. 2014). To the best of our knowledge, the application of a microcapsule agent as a convenient and effective remediation method for Cr(VI)-contaminated soil has not been investigated. In our lab culture experiments, the Cr(VI) reduction efficiency of strain ZC1 in the optimized medium was slightly lower than that in LB medium, which might be due to the rich nutrients contained in the LB medium (data not shown). However, in the simulated soil microcosm remediation experiments, Cr(VI) reduction was obviously enhanced with the addition of the microcapsule agent. Our results did not surpass the soil remediation efficiency reported previously for *B. cereus* YR5, which was able to completely remove nearly 100 % Cr(VI) within 30 days (Kumari et al. 2014). However, we achieved relatively high remediation efficiency with low cost and convenience for storage and transportation. Hence, the application of this microcapsule agent offers the potential of in situ bioremediation of Cr(VI)-contaminated soil. The next step is to apply the microcapsule agent of strain ZC1 in Cr(VI) bioremediation plant pot experiments.

## Conclusions

*Lysinibacillus fusiformis* ZC1 is a Cr(VI)-reducing bacterium with the potential to immobilize Cr(VI) in soil. In this study, we successfully produced a microcapsule agent of this strain. The microcapsule agent exhibited high viable cell counts, good stability and high efficiency in bioremediation of Cr(VI)-spiked soil. Hence, application of this microcapsule agent offers an important biological approach for treatment of Cr(VI)-contaminated soil.

## Methods

### Bacterial strain and optimization of culture medium

The bacterial strain used in this study was a highly efficient Cr(VI)-reducing bacterium known as *L. fusiformis* ZC1, which was isolated from the Cr-contaminated waste water of a metal electroplating factory in Guangdong Province, P. R. China (He et al. 2011). This bacterium is a Gram-positive rod-shaped strain that demonstrated a MIC of 60 mM to K_2_CrO_4_ and rapid Cr(VI) reduction in LB medium. Strain ZC1 was deposited in The Agricultural Research Service Culture Collection, USA (NRRL http://nrrl.ncaur.usda.gov) under the accession number NRRL B-59,453. In addition, the accession number of the entire genome sequence in the DDBJ/EMBL/GenBank is ADJR00000000.

To reduce the cost of the culture medium, single-factor experiments (Cochran and Cox 1957) were introduced in this study as a first optimization step to identify the best medium components. Strain ZC1 was separately inoculated into 100 mL original medium with various carbon sources (sucrose, corn flour, maltose, glucose, lactose, dextrin, starch), organic nitrogen sources (soya peptone, tryptone, soybean flour, yeast extract), inorganic nitrogen sources [KNO_3_, NH_4_Cl, CO(NH_2_)_2_, (NH_4_)_2_SO_4_] and inorganic salts (KH_2_PO_3_, NaCl, CaCl_2_, Na_2_SO_4_, KCl, MgSO_4_, K_2_HPO_3_) and incubated at 37 °C with 160 rpm shaking. After 30 h of cultivation, the numbers of cells and spores were counted following the method described by Chen et al. (2010). The optimal medium components were selected to maximize the cell and spore productions of strain ZC1. An orthogonal test (Lee et al. 1997) was performed to determine the best concentration of each component. In the orthogonal test, an experimental design of L_9_ (3^4^) was applied for four independent variables, each at three levels. The factors and their concentrations are shown in Table 1. Based on the orthogonal design, a total of nine experiments were performed simultaneously, and the orthogonal arrays are shown in Table 2. Data were analyzed by one-way analysis of variance (ANOVA) with SPSS 19.0 to identify the concentrations that had the most positive effect on strain ZC1 cell and spore productions.

### Bioreactor fermentation using the optimized medium

For production of the inoculum, one loopful of strain ZC1 culture was transferred from an agar slant to a 250 mL Erlenmeyer flask containing 50 mL LB medium. The culture was incubated on a rotary shaker at 37 °C with 160 rpm shaking for 18 h. Subculture was conducted by inoculating 2 mL overnight culture in a 500 mL Erlenmeyer flask containing 100 mL LB medium. The second-stage seed culture was incubated under the same conditions and used in inoculation of the fermenter. Batch fermentation was performed in a 50 L fermenter (BIOF-2000, China) with a sterilizable dissolved oxygen (DO) probe (Mettler Toledo) and a pH probe (Mettler Toledo). The DO and pH probes were both calibrated following standard procedures prior to sterilization. The DO probe was calibrated to zero and 100 % in saturated sodium sulfite solution and saturation air, respectively. The pH probe was calibrated to 7 in neutral standard solution (pH = 7) and adjusted to 4 in acidic standard solution (pH = 4). The optimized fermentation medium was sterilized in situ, and the fermentation was started in batch mode under the following conditions: medium volume of 30 L, inoculum volume of 2 % (v/v), temperature of 37 °C and pH of 8.0. In addition, the aeration and agitation were controlled to maintain the dissolved oxygen percentage above 50 % saturation during the fermentation process, and 0.04 % (v/v) dimethyl silicone oil was used as an antifoaming agent. Sampling was performed aseptically every 4 h for determination of the cell and spore yields. The fermentation was stopped at 28 h in the early-stationary phase.

### Production of microcapsule agent of strain ZC1 cells by spray drying and storage

Feed solutions were prepared following the method described by Lian et al. (2002). Bacterial cells from the 30 L fermentation broth were harvested by centrifugation (8000 rpm for 10 min at 4 °C) and resuspended in 6 L PBS buffer (0.2 M, pH = 7.5) after fermentation. To obtain the best protective agent, different carriers were investigated, including gelatin, gelatin:sorbitol (1:1), gelatin:sucrose (1:1), gelatin:starch (1:1), gum arabic, gum arabic:sorbitol (1:1), gum arabic:sucrose (1:1) and gum arabic:starch (1:1). The cell suspension was mixed with these carrier solutions [3 % (w/v)] at a ratio of 2:1 (v/v) and vigorously shaken with a vortex mixer for 20 min. Spray drying of *L. fusiformis* ZC1 cells mixed with various carrier solutions was performed in a laboratory scale spray dryer (SD-1500, China). The spray-drying conditions were inlet air temperature of 160 °C, outlet air temperature of 70–75 °C and atomizing air pressure of 0.3 MPa. The powders were collected in a single cyclone separator. The spray-dried microcapsule agent particles were stored in sealed bags made of aluminum–polyester–polyethylene at 4 °C and room temperature. To determine the survival rate of the cells in the microcapsule agent, the particles were incubated for 50 days, and samples were periodically collected for viable plate counts. The sample processing method was described by Gardiner et al. (2000).

### Scanning electron microscopy (SEM) observation

The external morphology of the spray-dried microcapsule agent particles was observed using a scanning electron microscope (SEM; JSM-6390, JEOL, Japan). Prior to microscopy observation, the samples were mounted on aluminum stubs and covered with gold using an ion-sputtering device (JFC-1600, JEOL, Japan). Images of the particles were viewed with an acceleration voltage of 20 kV.

### Soil microcosm Cr(VI) remediation experiment

The soil was collected from a woodland area at Huazhong Agricultural University (Wuhan, Hubei Province, China). After air drying for 7 days, the soil was ground and sieved through a 1 mm screen and mixed thoroughly with Cr(VI) to an initial concentration of 28 mg/kg dw soil. For microcapsule agent treatment, the spray-dried particles were suspended in PBS buffer (0.2 M, pH = 7.5) and added to each container with 60 g soil (triplicates) to a final concentration of 10^8^ cfu/g dw soil. The same soils without the addition of a microcapsule agent were used as controls. The differently treated soils were incubated in the dark at 30 °C for 30 days, and the moisture of the soils was maintained at 16 % throughout the experiment via periodic watering. Soil samples were periodically collected for determination of Cr(VI) concentration using the USEPA Method 3060A described by Xiao et al. (2012). Fresh soils (1.25 g) were digested with 25 mL 0.28 mol/L Na_2_CO_3_ and 0.5 mol/L NaOH in a 50 mL digestion tube. The solutions were heated at 95 °C for 60 min with continuous stirring. After cooling, the digested suspension was filtered and metered to a volume of 25 mL with ddH_2_O. The pH values of the filtrates were adjusted to between 7 and 8 with nitric acid, and the samples were centrifuged (8000 rpm for 10 min) to remove the precipitation. The concentrations of Cr(VI) in the supernatants were analyzed using the diphenylcarbohydrazide (DPC) spectrophotometric method (Monteiro et al. 2002).
